# Supplementary material for: A mouse model of autosomal dominant spastic ataxia and myopathy caused by a mutation in Tuba4a
Source: Hum Mol Genet. 2026 Sep 1;35(18):ddag084. doi: 10.1093/hmg/ddag084 (PMC13626205; doi:10.1093/hmg/ddag084)
Supplement: Supplementary file 1 [file supplementary_materials_ddag084.zip › Supplemental materials 27July2026.pdf]

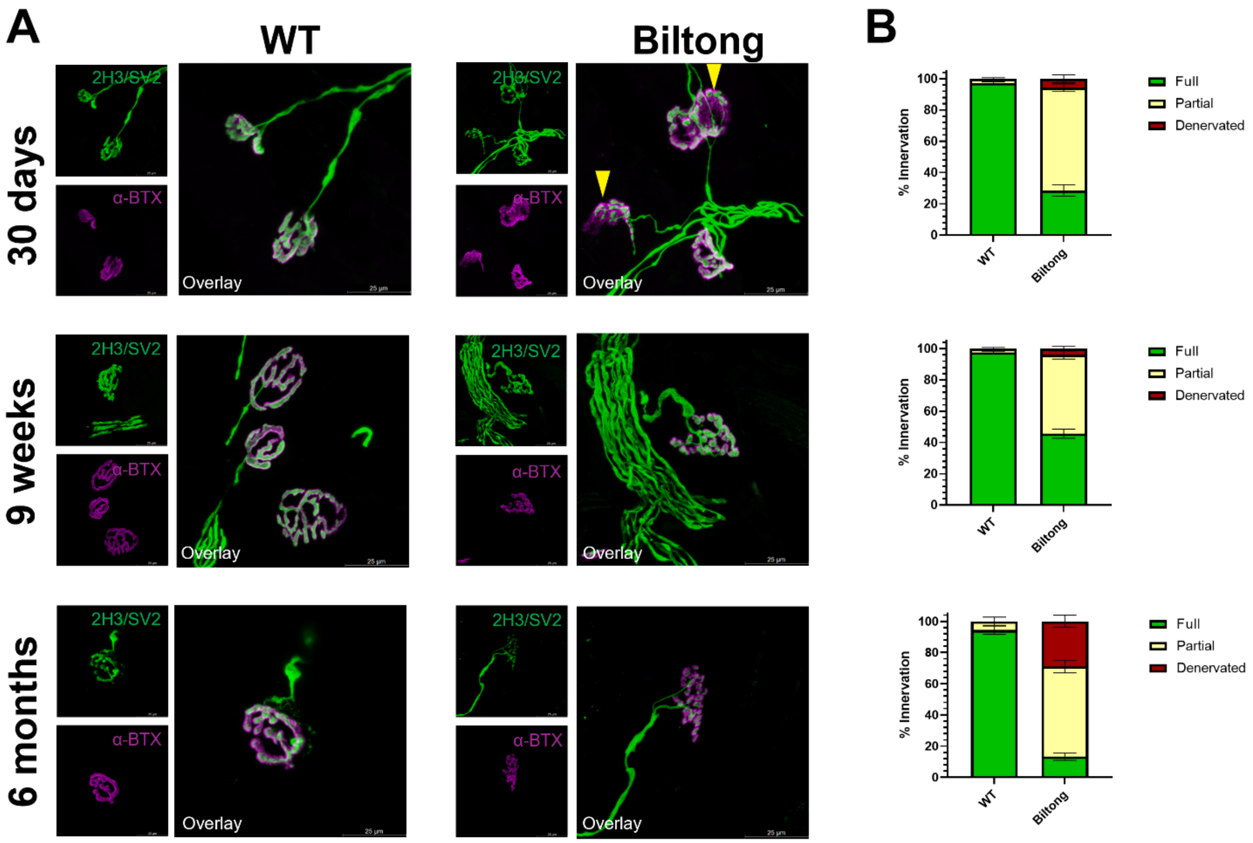


**Supplementary Figure 1.** NMJ denervation in plantaris of Biltong mice. **A)** Images of plantaris NMJs from WT and Biltong mice at three timepoints (30d, 9wk, and 6mo). The axon terminal/presynapse is labeled in green with a cocktail of SV2 and NFM antibodies, while the acetylcholine receptors/postsynapse are labeled in magenta with AlexaFluor594-conjugated α-bungarotoxin. Yellow arrowheads in 30 d Biltong image indicate partially innervated NMJs. Scale bars are 25 µm. **B)** Quantification of fully innervated (green), partially innervated (yellow), and denervated (red) NMJs from the same mice as in (A). N = 3 WT, 7 Biltong at 30 d; 10 WT, 11 Biltong at 9 wk; and 10 WT, 13 Biltong at 6 mo.

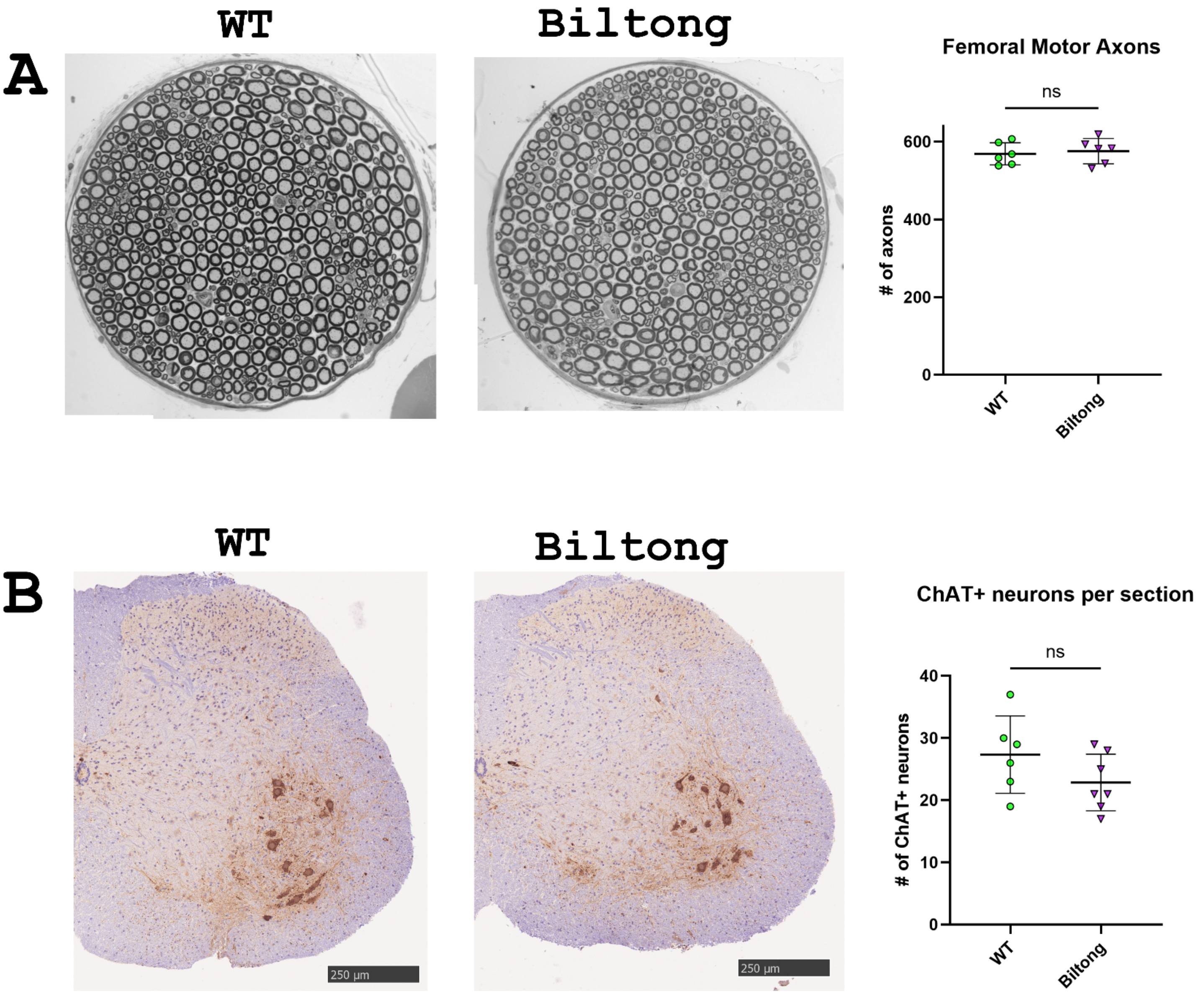


**Supplementary Figure 2.** Biltong mice do not show signs of motor neuron degeneration at 6 months. **A)** Images of semithin toluidine blue-stained cross-sections of the motor branch of the femoral nerve and quantification of the number of axons in nerves from WT and Biltong mice at 6 months. N = 6 WT and 6 Biltong mice. **B)** Images of ChAT-labeled spinal cord cross-sections and quantification of motor neurons in spinal cord sections from WT and Biltong mice at 6 months. N = 6 WT and 7 Biltong mice.

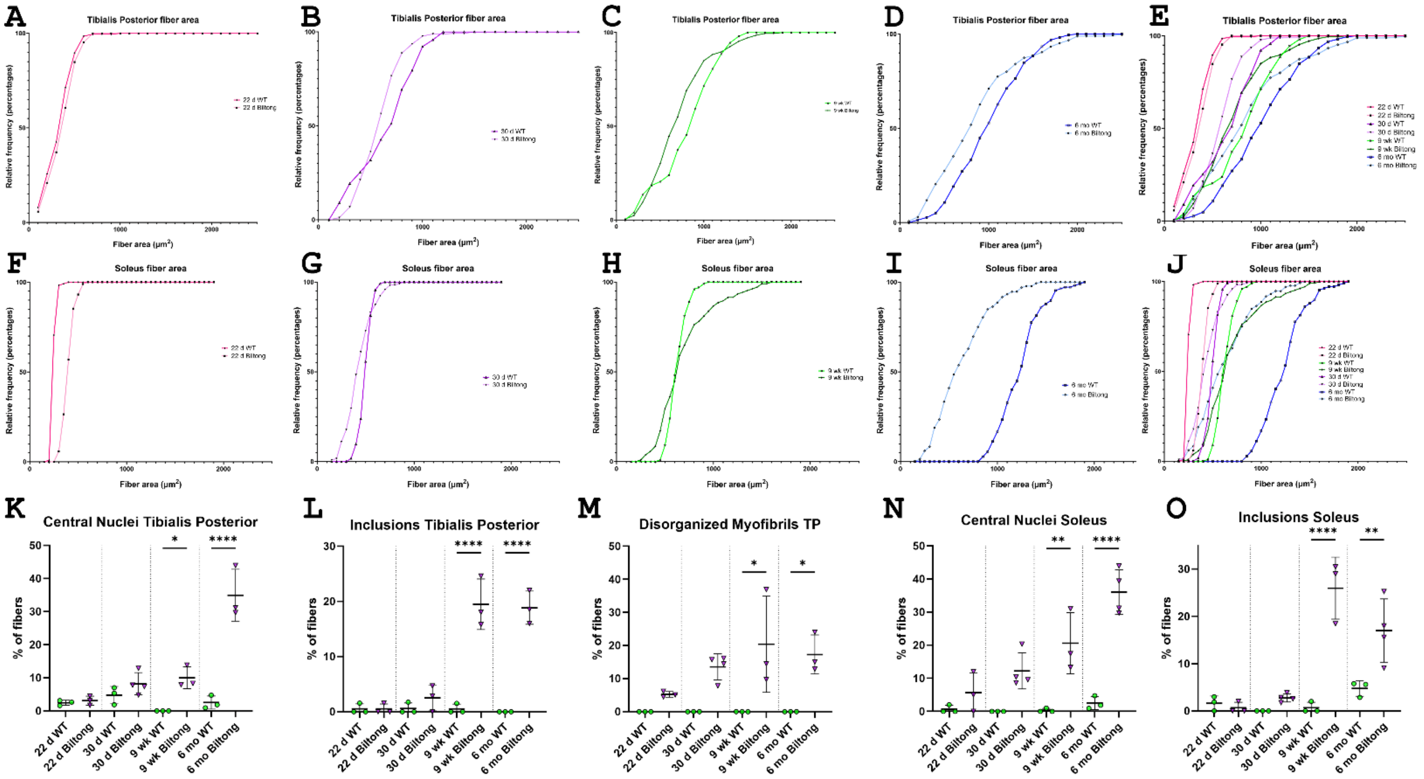

**Supplementary Figure 3.** Quantification of skeletal muscle fiber size and pathology in the soleus and tibialis posterior. **A – D)** Cumulative frequency distribution plots of muscle fiber area in tibialis posterior from WT and Biltong mice at 22 d, 30 d, 9 wk, and 6 mo, respectively. **E)** Combined cumulative frequence distribution plot with all timepoints in A – D. **F – I)** Cumulative frequency distribution plots of muscle fiber area in soleus from WT and Biltong mice at 22 d, 30 d, 9 wk, and 6 mo, respectively. **J)** Combined cumulative frequency distribution plot with all timepoints in F – I. **K – M)** Percentage of muscle fibers in tibialis posterior with central nuclei, inclusions, or disorganized myofibrils, respectively. **N & O)** Percentage of muscle fibers in soleus with central nuclei or inclusions, respectively. TP = tibialis posterior. * $p < 0.05$, ** $p < 0.01$, **** $p < 0.0001$ by one-way ANOVA.

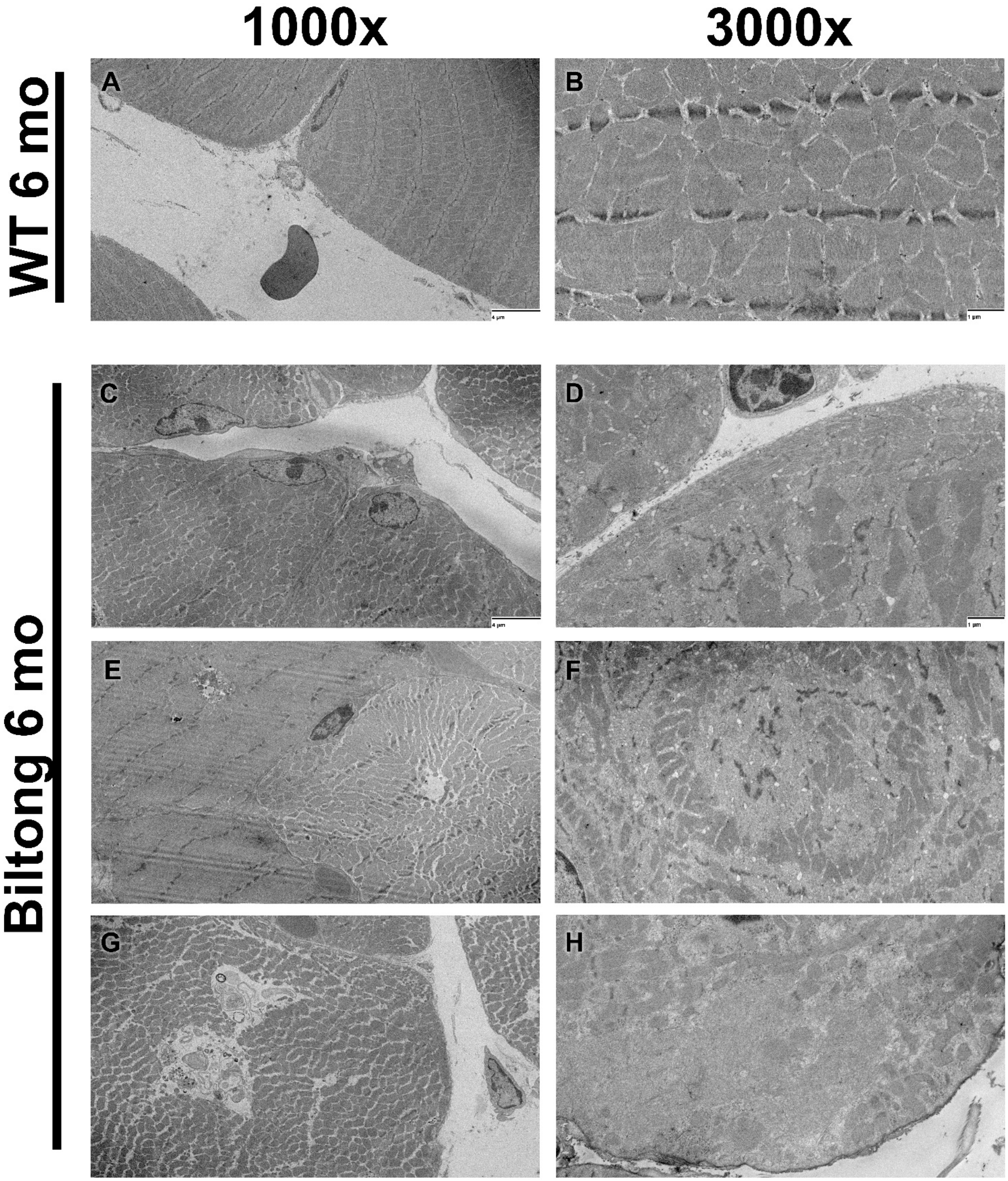


**Supplementary Figure 4.** No true ringbinden seen with TEM on tibialis posterior from Biltong mice. **A-B)** Representative TEM images of tibialis posterior muscle from WT mice showing normal ultrastructure. **C-H)** TEM images of tibialis posterior muscle from Biltong mice revealed pathology similar to the soleus (Figure 6) including Z-band streaming, osmiophilic inclusions and multilamellar myeloid bodies, and numerous fibers with disorganized myofibrils. However, no fibers were seen with the typical perpendicular re-organization of myofibrils seen in true ringbinden observed in myotonic or limb-girdle muscular dystrophy patients.

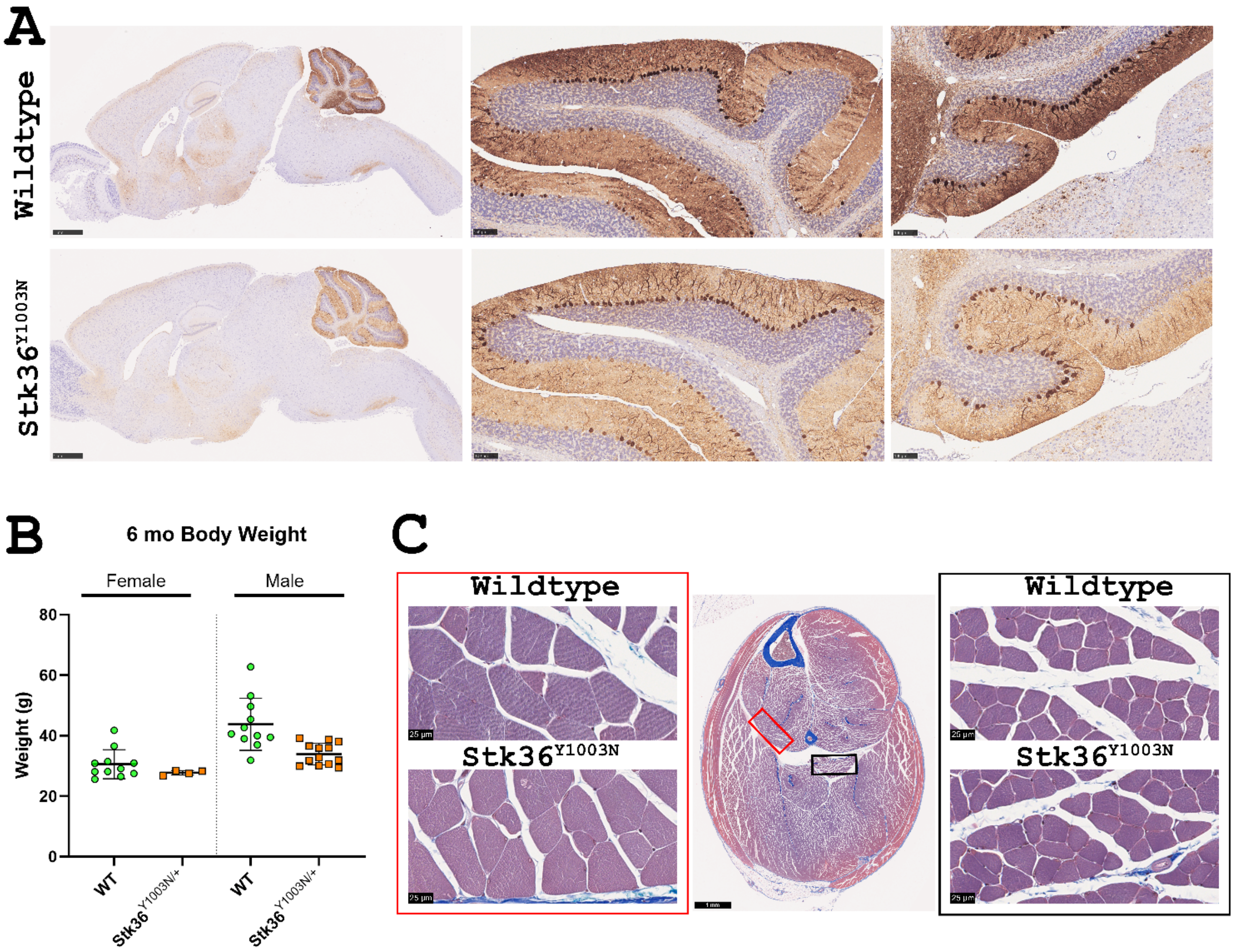


**Supplementary Figure 5.** *Stk36*$^{Y1003N}$ knockin mouse does not produce a discernible phenotype by 6 months. **A)** Sagittal brain sections from 6 mo WT and *Stk36*$^{Y1003N}$ knockin mice stained with Calbindin to label cerebellar Purkinje neurons. High magnification images show lobules VI (left) and X (right). Scale bars are 1 mm (whole brain) and 100 µm (lobules VI and X). **B)** Body weights from 6 mo WT and *Stk36*$^{Y1003N}$ female and male mice. **C)** Masson's trichrome staining of lower hindlimb cross-sections taken from WT and *Stk36*$^{Y1003N}$ knockin mice at 6 months. High magnification (80x) insets are shown from the tibialis posterior (left, red rectangle) and soleus (right, black rectangle). Scale bars are 1 mm (whole hindlimb section) or 25 µm (80x images).

**Movie 1.** Biltong mice have severe ataxia and muscle wasting. Movie of a 6 mo Biltong mouse (white) with an unaffected littermate (brown) shows the decreased body weight and shaky, uncoordinated movement caused by the mutation.

**Movie 2.** Biltong mice have severe ataxia and muscle wasting. Movie of a 6 mo Biltong mouse (front) with an unaffected littermate (back) shows the motor deficits and decreased body weight caused by the mutation.

**Movie 3.** *Tuba4a*$^{Q176P}$ CRISPR mice recapitulate ataxia and muscle wasting phenotypes seen in Biltong mice. Movie of a 5 mo *Tuba4a*$^{Q176P}$ CRISPR mouse (starts at left) with an unaffected littermate (starts at right) shows the decreased body weight and shaky, uncoordinated movement caused by the mutation.

**Movie 4.** *Tuba4a*$^{Q176P}$ CRISPR mice recapitulate ataxia and muscle wasting phenotypes seen in Biltong mice. Movie of a 4 mo *Tuba4a*$^{Q176P}$ CRISPR mouse (starts at right) with an unaffected littermate (starts at left) shows the motor deficits and decreased body weight caused by the mutation.

**Table 1.** Mouse and human α- and β-tubulin genes.

| | **Mouse** | **Human** |
|---|---|---|
| **α-tubulin genes** | *Tuba1a* | *TUBA1A* |
| | *Tuba1b* | *TUBA1B* |
| | *Tuba1c* | *TUBA1C* |
| | *Tuba3a* | - |
| | *Tuba3b* | - |
| | - | *TUBA3C* |
| | - | *TUBA3D* |
| | - | *TUBA3E* |
| | *Tuba4a* | *TUBA4A* |
| | - | *TUBA4B* |
| | *Tuba8* | *TUBA8* |
| | *Tubal3* | *TUBAL3* |
| **β-tubulin genes** | - | *TUBB* |
| | *Tubb1* | *TUBB1* |
| | *Tubb2a* | *TUBB2A* |
| | *Tubb2b* | *TUBB2B* |
| | *Tubb3* | *TUBB3* |
| | *Tubb4a* | *TUBB4A* |
| | *Tubb4b* | *TUBB4B* |
| | *Tubb5* | - |
| | *Tubb6* | *TUBB6* |
| | - | *TUBB8* |

**Abbreviations:**

ENU: N-ethyl-N-nitrosourea
SNP: single nucleotide polymorphism
CMYO: congenital myopathy
SPAX: spastic ataxia
FTD: frontotemporal dementia
ALS: amyotrophic lateral sclerosis
CRISPR: clustered regularly interspaced short palindromic repeats
Bp: base pairs
WES: whole exome sequencing
WGS: whole genome sequencing
EMG: electromyography
CMAP: compound muscle action potential
NCV: nerve conduction velocity
RNS: repetitive nerve stimulation
WT: wild-type
NMJ: neuromuscular junction
CV/LFB: cresyl violet luxol fast blue
PN: Purkinje neuron
TEM: transmission electron microscopy
IVF: in vitro fertilization
MRI: magnetic resonance imaging
PD: Parkinson's disease
PCR: polymerase chain reaction
DNA: deoxyribonucleic acid
PVDF: polyvinylidene difluoride
PBS-T: phosphate buffered saline with Tween 20
HRP: horseradish peroxidase
NBF: neutral buffered formalin
PFA: paraformaldehyde
BSA: bovine serum albumin
